# Infection Prevention and Control Content Inclusion in Graduate Public Health Curricula

**DOI:** 10.1017/ash.2025.208

**Published:** 2025-09-24

**Authors:** Lorelei Herman, Arabel Severe, Christine McGuire-Wolfe, USF Infection Control for Emergency Responders

**Affiliations:** 1 University of South Florida College of Public Health; 2 USF College of Public Health; 3 University of South Florida - College of Public Health

## Abstract

The field of public health is facing greater demand, significant staff turnover, and an increasing number of public health emergencies and threats. This is further compounded by an unprecedented unmet need for infection preventionists (IPs) in the workforce. The integration of infection prevention and control (IPC) material into existing public health (PH) academic programming could bridge this gap. There are very few IPC-concentrated Masters of Public Health (MPH) programs and the extent of IPC focused content in existing graduate PH programs is unknown. This project seeks to define the extent to which graduate public health courses include IPC concepts and identify potential inclusion points for these topics.

Syllabi for core PH courses were requested from all Council on Education for Public Health (CEPH) accredited graduate schools, of which there were 137 at the time of retrieval. Received syllabi (n = 245) were reviewed and coded for inclusion of IPC topics such as antibiotic resistance and antibiotic stewardship (AR) and healthcare acquired infections (HAIs). These syllabi represented 54 programs (39%) and 34 states. An additional six (6) states had no applicable programs.

Seventy-six (31%) syllabi had specific IPC content, while an additional 119 (49%) had potential inclusion points for IPC content. Seventy-two courses (30%) had neither IPC content nor potential inclusion points; these courses tended to be biostatistics, health policy and management, or environmental health classes. All analyzed MPH academic programs had at least one area within the core courses that served as a potential inclusion point for IPC content, supporting the argument that public health core competencies naturally align with IPC domains outlined in the Association for Professionals in Infection Control and Epidemiology (APIC) Infection Preventionist competency model.

Observations from this review indicate both the capability to seamlessly integrate IPC material into MPH programs and the existing deficit where this opportunity is unrealized. These findings can guide the development of tool kits to integrate the outlined inclusion points into existing graduate public health curricula guiding future workforce development to address current limitations.

